# An Assessment of Physical Activity Data Collected via a Smartphone App and a Smart Band in Breast Cancer Survivors: Observational Study

**DOI:** 10.2196/13463

**Published:** 2019-09-06

**Authors:** Il Yong Chung, Miyeon Jung, Sae Byul Lee, Jong Won Lee, Yu Rang Park, Daegon Cho, Haekwon Chung, Soyoung Youn, Yul Ha Min, Hye Jin Park, Minsun Lee, Seockhoon Chung, Byung Ho Son, Sei-Hyun Ahn

**Affiliations:** 1 Department of Surgery Asan Medical Center University of Ulsan College of Medicine Seoul Republic of Korea; 2 College of Business Korea Advanced Institute of Science and Technology Seoul Republic of Korea; 3 Department of Biomedical Systems Informatics Yonsei University College of Medicine Seoul Republic of Korea; 4 Swallaby Co, Ltd Seoul Republic of Korea; 5 Department of Psychiatry Asan Medical Center University of Ulsan College of Medicine Seoul Republic of Korea; 6 College of Nursing Gachon University Incheon Republic of Korea

**Keywords:** telemedicine, breast neoplasms, mobile apps, quality of life, stress, psychological, patient compliance, smartphone, mobile phone, wearable electronic devices, survivorship

## Abstract

**Background:**

Although distress screening is crucial for cancer survivors, it is not easy for clinicians to recognize distress. Physical activity (PA) data collected by mobile devices such as smart bands and smartphone apps have the potential to be used to screen distress in breast cancer survivors.

**Objective:**

The aim of this study was to assess data collection rates of smartphone apps and smart bands in terms of PA data, investigate the correlation between PA data from mobile devices and distress-related questionnaires from smartphone apps, and demonstrate factors associated with data collection with smart bands and smartphone apps in breast cancer survivors.

**Methods:**

In this prospective observational study, patients who underwent surgery for breast cancer at Asan Medical Center, Seoul, Republic of Korea, between June 2017 and March 2018 were enrolled and asked to use both a smartphone app and smart band for 6 months. The overall compliance rates of the daily PA data collection via the smartphone walking apps and wearable smart bands were analyzed in a within-subject manner. The longitudinal daily collection rates were calculated to examine the dropout pattern. We also performed multivariate linear regression analysis to examine factors associated with compliance with daily collection. Finally, we tested the correlation between the count of daily average steps and distress level using Pearson correlation analysis.

**Results:**

A total of 160 female patients who underwent breast cancer surgeries were enrolled. The overall compliance rates for using a smartphone app and smart bands were 88.0% (24,224/27,513) and 52.5% (14,431/27,513), respectively. The longitudinal compliance rate for smartphone apps was 77.8% at day 180, while the longitudinal compliance rate for smart bands rapidly decreased over time, reaching 17.5% at day 180. Subjects who were young, with other comorbidities, or receiving antihormonal therapy or targeted therapy showed significantly higher compliance rates to the smartphone app. However, no factor was associated with the compliance rate to the smart band. In terms of the correlation between the count of daily steps and distress level, step counts collected via smart band showed a significant correlation with distress level.

**Conclusions:**

Smartphone apps or smart bands are feasible tools to collect data on the physical activity of breast cancer survivors. PA data from mobile devices are correlated with participants’ distress data, which suggests the potential role of mobile devices in the management of distress in breast cancer survivors.

**Trial Registration:**

ClinicalTrials.gov NCT03072966; https://clinicaltrials.gov/ct2/show/NCT03072966

## Introduction

Distress screening is important in cancer survivorship care. The prevalence of depression and anxiety were reported to be high among cancer survivors [1,2]. The National Comprehensive Cancer Network and American Society of Clinical Oncology guidelines recommend all cancer patients be screened for depressive symptoms [3,4].

Although distress screening is crucial for cancer survivors, it is not easy for clinicians to recognize distress. Some studies have demonstrated that the prevalence and severity of depression are underestimated [4,5]. Moreover, conventional screening tools for distress are patient-reported outcomes (PROs) based on paper questionnaires, which are subject to recall bias and do not reflect real-time episodes of distress.

Electronic PROs (ePROs) using a smartphone app can be used as a screening tool for depression in practice [6]. While the completion rate of online questionnaires in cancer patients was about 15.0% from home, the overall compliance rate increased when patients used smartphone apps [7-9]. Although ePROs using apps is a feasible tool for distress screening, there is still room for improvement in adherence rates.

Researchers have recently suggested that changes in physical activities (PA) per the data collected by wearable devices can predict mood changes [10-11]. PA data collected by mobile devices such as smart bands and smartphone apps to screen distress in cancer survivors could offer tremendous advantages. However, the previous studies were not conducted in cancer survivors, the sample size was relatively small, and distress or depression was not evaluated by conventional screening tools.

The prerequisite and essential condition for using mobile devices for data collection is compliance to mobile devices. However, long-term compliance to mobile devices, especially in clinical setting, has rarely been studied. According to a systematic review [12], among compliance studies with mobile device–based ecological momentary assessment, about half of the studies (24/42, 62%) recruited youth from nonclinical settings, the length of studies ranged only from 2 to 42 days, and the sample size in clinical settings was small (range 5 to 84 participants). Until now, there has been a lack of evidence about long-term compliance and data collection using mobile devices.

We conducted a single-center, prospective, observational study with 160 breast cancer patients who underwent breast cancer surgery and 6-month follow-up. The aim of this study was to assess data collection rates of smartphone apps and smart bands in terms of PA data to investigate the correlation between PA data from mobile devices and distress-related questionnaire data from smartphone apps and demonstrate factors associated with data collection with smart bands and smartphone apps in breast cancer survivors.

## Methods

### Study Setting and Subjects

This prospective observational study recruited patients who underwent surgeries for breast cancer at Asan Medical Center, Seoul, Republic of Korea. Patients were eligible for study participation if they were women between the ages of 20 and 65 years and had their own Android smartphones compatible with the WalkOn app (Swallaby Co, Ltd), a free activity tracking app modified for this study. Patients who had distant metastasis, recurrent breast cancer, or severe medical conditions such as cardiovascular disease and those who had no capability of using a smartphone were excluded. Patients on chemotherapy were also excluded.

Written informed consent was obtained from all subjects at enrollment. The study protocol was approved by the institutional review board at Asan Medical Center (2016-0819). This study was registered on the ClinicalTrial.gov website (NCT03072966).

### Recruitment and Follow-Up

During the hospital stay after breast cancer surgery, subjects were contacted by a clinical research assistant. After consenting to participate, subjects completed paper-based questionnaires (Distress Thermometer [DT] and Patient Health Questionnaire–9 [PHQ-9]) at baseline. The clinical research assistant helped to download the Android-based app (WalkOn) to the participants’ smartphones.

At the first visit to the clinic after discharge, subjects who are not undergoing adjuvant chemotherapy received wearable devices (Fitbit Charge HR, Fitbit Inc), and the Fitbit apps were installed on their smartphones. Subjects scheduled to receive adjuvant chemotherapy were provided the wearable devices after chemotherapy. The clinical research assistant called participants after 3 weeks to follow up and assist with collection of data from the wearable devices. At the 3- and 6-month follow-ups, participants’ smartphone apps and smart bands were checked by the clinical research assistant.

### Smartphone App and App-Based Questionnaire

The health-related smartphone app, WalkOn, has been developed by the mobile phone health care app company, Swallaby Co, Ltd. The app provides users with platforms for tracking their daily steps and creates mobile communities where users can communicate with each other and view other members’ daily steps to motivate them and promote health-related activities.

App-based self-reporting questionnaires have been included in this app, which exclusively allows study participants to complete daily, weekly, and biweekly questionnaires. The daily questionnaires developed and previously reported by the authors consisted of self-reporting modules for daily anxiety, sleep, and emotion [6,9]. Weekly DT and biweekly PHQ-9 questionnaires were also collected through the app, and push alarms from Sunday to Tuesday every week were sent to subjects’ smartphones as notification.

### Activity Tracking From Smartphone Apps and Smart Bands

We used the app to collect data on participants’ daily steps. Once the app was activated at enrollment, participants were instructed to open the app and pull-to-refresh the front page of the app at least once weekly to send the weekly bundle of daily walking data to a central database system. The central database system collected the daily walking data for each subject with anonymized user ID, item ID, date and time of input, and input value.

As a second channel for data collection, a smart band (Fitbit Charge HR) was also used to monitor daily PA and sleep patterns in real time during the 6-month study period. Briefly, the Fitbit Charge HR is designed to measure various PAs such as steps taken, distance traveled, and calories burned. It also shows users how many minutes they have been active during the day. Similar to the walking app, after Fitbit activation, participants were instructed to access the Fitbit app and pull-to-refresh at least once per week to send the weekly set of daily walking data to the server. The Fitbit app also sent a push notification to the users about changes in daily activity patterns. While the mobile walking app only collects the number of steps per day, the Fitbit collects various indicators about PA each minute. The central database system collected all indicators at both minute- and day-levels using anonymized user ID, item ID, date and time of input, and input values.

### Statistical Analysis

The feasibility of the app-based and smart band–based PA collecting systems were analyzed by calculating individual-level data collection rates, defined as the total number of days in which data collection was completed divided by the number of follow-up days for each patient as well as a longitudinal day-level data collection rate, defined as the total number of patients with PA data divided by the number of patients who did not drop out on a single specific day from the start of data collection. The cumulative longitudinal day-level data collection rate was defined as the mean of all longitudinal day-level data collection rates from the starting day until the specified day. For example, the longitudinal day-level collection rate at day 20 indicated the number of patient data collection points on the 20th day divided by 160, while the cumulative longitudinal day-level data collection rate at 20th day was the mean value of all longitudinal day-level data collection rates from day 0 to day 20. The definitions of individual-level, longitudinal day-level, and cumulative longitudinal day-level data collection rates were similar with the measurements of compliance which were described previously [9].

Student *t* tests and chi-square tests were used to investigate the differences in patient characteristics between high and low data collection rate groups. The variables of interest included age, marital status, education, occupation, comorbidity, past episode of depression, surgery, chemotherapy, radiation therapy, antihormonal therapy, targeted therapy, and stage. The mean value of individual-level data collection rates was used as the cutoff to divide the high and low collection rate groups. The mean individual-level rates were 91.1% for the smartphone app and 55.0% for the smart band.

The Pearson correlation test was used to calculate the correlation between app-based and band-based collection rates. To more comprehensively investigate the factors affecting individual-level collection rates, we conducted multivariate linear regression analysis for both app- and band-based data collection rates with all variables. Since two patients did not report baseline distress levels in presurvey, we exclude them from step 2 and 3 analyses.

Finally, we examined the correlation between app-based and band-based step counts and distress-related psychological questionnaires using Pearson correlation tests.

## Results

### Descriptive Statistics

**Figure 1:**
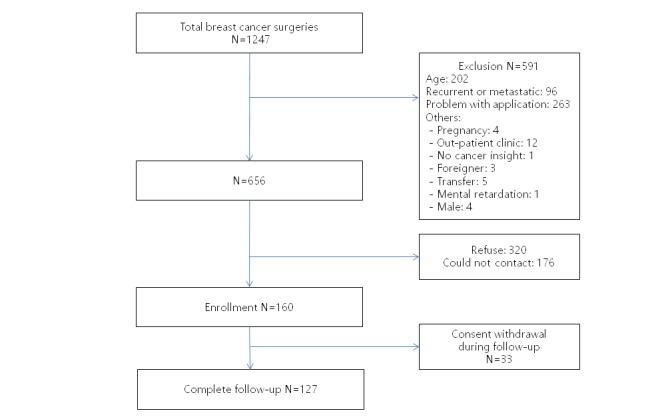
Subject enrollment.

From June 2017 to January 2018, we assessed for study eligibility 1247 consecutive patients who underwent breast cancer surgery (Figure 1). After screening, 591 patients were excluded; 176 patients were not able to be contacted during the hospital stay and 320 patients refused to join the study. A total of 160 patients were enrolled in this study. During follow-up, 33 participants withdrew consent due to inconvenience (n=26), transfer to other hospitals (n=2), app compatibility problems after update (n=4), and breast cancer recurrence (n=1).

Table 1 summarizes descriptive statistics of the demographic and clinical characteristics of the subjects as absolute and relative frequencies. Subjects were aged mean 45.3 [SD 6.8] years (range 28 to 64 years), and about three-quarters (121/160, 75.6%) were younger than 50 years. Sixty-four subjects completed adjuvant or neoadjuvant chemotherapy before beginning daily data collection. Among the 160 patients, 75.6% (121/160) were less than 50 years of age, 66.9% (107/160) had an educational attainment of college level or higher, and 47.5% (76/160) were currently employed. The mean baseline EQ5D-5L score was 0.91 (SD 0.1). Regarding breast cancer stages, 13.1% (21/160) of patients had stage 0, 48.1% (77/160) had stage I, 24.4% (39/160) had stage II, and 14.4% (23/160) had stage III disease.

**Table 1:** Subject demographics.

Characteristics		Total (N=160)	App compliance							Band compliance							
			Higher (n=133)	Lower (n=27)		*P* value			Higher (n=80)			Lower (n=80)			*P* value		
**Age (years), mean (SD)**		**45.3 (6.8)**	**44.9 (6.9)**	**47.1 (6.4)**		**.13**			**44.9 (6.5)**			**45.7 (7.1)**			**.43**		
	<50, n (%)	121 (75.6)	103 (77.4)	18 (66.7)		.35			65 (81.3)			56 (70.0)			.14		
	≥50, n (%)	39 (24.4)	30 (22.6)	9 (33.3)		—^a^			15 (18.7)			24 (30.0)			—		
**Marital status, n (%)**						**.63**									**.73**		
	Married	137 (85.6)	114 (85.7)	23 (85.2)					70 (87.5)			67 (83.8)					
	Single	20 (12.5)	17 (12.8)	3 (11.1)					9 (11.3)			11 (13.7)					
	Other	3 (1.9)	2 (1.5)	1 (3.7)					1 (1.2)			2 (2.5)					
**Education, n (%)**						**.49**									**.74**		
	≤High school	53 (33.1)	42 (31.6)	11 (40.7)					25 (31.3)			28 (35.0)					
	>High school	107 (66.9)	91 (68.4)	16 (59.3)					55 (68.7)			52 (65.0)					
**Occupation, n (%)**						**.33**									**.43**		
	Yes	76 (47.5)	66 (49.6)	10 (37.0)					41 (51.3)			35 (43.8)					
	No	84 (52.5)	67 (50.4)	17 (63.0)					39 (48.8)			45 (56.2)					
**Comorbidity, n (%)**						**.03**									**.24**		
	Yes	108 (67.5)	95 (71.4)	13 (48.1)					50 (62.5)			58 (72.5)					
	No	52 (32.5)	38 (28.6)	14 (51.9)					30 (37.5)			22 (27.5)					
**Past episode of depression, n (%)**							**.498**									**.84**	
	Yes	2 (1.3)	2 (1.5)	0 (0)					1 (1.3)			1 (1.3)					
	No	152 (95.0)	127 (95.5)	25 (92.6)					77 (96.3)			75 (93.7)					
	No response	6 (3.8)	4 (3.0)	2 (7.4)					2 (2.4)			4 (5.0)					
**Surgery, n (%)**						**.60**									**.08**		
	Mastectomy	11 (6.9)	9 (6.8)	2 (59.3)					7 (8.8)			4 (5.0)					
	Breast-conserving surgery	107 (66.9)	91 (68.4)	16 (7.4)					58 (72.5)			49 (61.3)					
	Mastectomy with reconstruction	42 (26.3)	33 (24.8)	9 (33.3)					15 (18.8)			27 (33.7)					
**Previous chemotherapy, n (%)**							**.28**									**.33**	
	Yes	65 (40.6)	51 (38.3)	14 (51.9)					36 (45.0)			29 (36.3)					
	No	95 (59.4)	82 (61.7)	13 (48.1)					44 (55.0)			51 (63.7)					
**Antihormonal therapy, n (%)**							**.04**									**>.99**	
	Yes	135 (84.4)	116 (87.2)	19 (70.4)					68 (85.0)			67 (83.7)					
	No	25 (15.6)	17 (12.8)	8 (29.6)					12 (15.0)			13 (16.3)					
**Radiation therapy, n (%)**						**.47**									**.34**		
	Yes	124 (77.5)	105 (78.9)	19 (70.4)					65 (81.3)			59 (73.8)					
	No	36 (22.5)	28 (21.1)	8 (29.6)					15 (18.7)			21 (26.3)					
**Targeted therapy, n (%)**						**.01**									**.76**		
	Yes	148 (92.5)	127 (95.5)	21 (77.8)					73 (91.3)			75 (93.8)					
	No	12 (7.5)	6 (4.5)	6 (22.2)					7 (8.7)			5 (6.2)					
**Stage, n (%)**						**.31**									**.42**		
	0	21 (13.1)	16 (12.0)	5 (18.5)					8 (10.0)			13 (16.3)					
	I	77 (48.1)	68 (51.1)	9 (33.3)					39 (48.8)			38 (47.5)					
	II	39 (24.4)	30 (22.6)	9 (33.3)					23 (28.7)			16 (20.0)					
	III	23 (14.4)	19 (14.3)	4 (14.8)					10 (12.5)			13 (16.3)					
**Distress thermometer score, n (%)**								**.29**									**.32**
	≥5	59 (36.9)	46 (34.6)	13 (48.1)					26 (32.5)			33 (41.3)					
	≤5	99 (61.9)	85 (63.9)	14 (51.9)					53 (66.3)			46 (57.5)					
	No response	2 (1.2)	2 (1.5)	0 (0)					1 (1.2)			1 (1.2)					
**HRQOL^b^ with EQ5D-5L^c^**						**.14**									**.44**		
	EQ5D-5L index, mean (SD)	0.91 (0.1)	0.92 (0.1)	0.87 (0.1)					0.92 (0.1)			0.90 (0.1)					
	No response, n (%)	2 (1.2)	2 (1.5)	0 (0)					1 (1.2)			1 (1.2)					
**PHQ-9^d^ total score, n (%)**						**.93**									**.71**		
	≥11	37 (23.2)	30 (22.6)	7 (25.9)					20 (25.0)			17 (21.3)					
	≤11	121 (75.6)	101 (75.9)	20 (74.1)					59 (73.8)			62 (77.5)					
	No response	2 (1.2)	2 (1.5)	0 (0)					1 (1.2)			1 (1.2)					
Median follow-up in days, median (SD)		164 (39.3)	164 (39.3)	164 (40.4)		.95			160 (46.6)			168 (30.2)			.18		

^a^Not applicable.

^b^HRQOL: Health-Related Quality of Life.

^c^EQ5D-5L: 5-dimension 5-level health questionnaire.

^d^PHQ-9: Patient Health Questionnaire–9.

### Data Collection Rates

Among 160 participants, 127 completed the 180-day study period, and 33 patients had different follow-up periods depending on the withdrawal date (minimum: 11 days, maximum: 176 days, median: 101 days). The total sum of follow-up days of all participants is 27,513, slightly less than 28,800 (160*180) because of patient dropout.

In total, 24,224 and 14,431 data points were collected via smartphone apps and smart bands, respectively. The overall data collection rates for using the smartphone app and smart band were 88.05% (24,224/27,513) and 52.45% (14,431/27,513), respectively.

**Figure 2:**
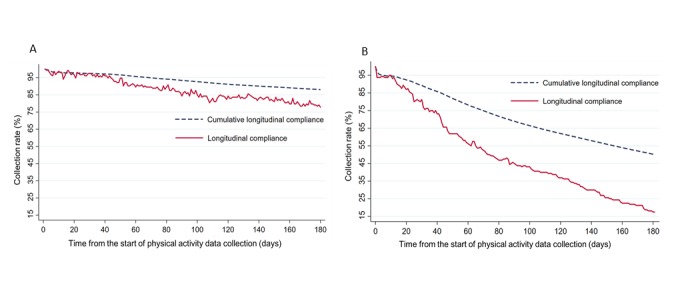
Longitudinal day-level data collection rates: (A) smartphone app; (B) smart band.

The data collection rate via smartphone app was >80% for 141 subjects (88.1%) and via smart band was >80% for 53 participants (33.1%). The longitudinal day-level data collection rates from day 1 to day 180 were calculated at daily intervals and plotted (Figure 2). For the smartphone app, the longitudinal data collection rate remained above 75%, reaching 77.8% at day 180, while the cumulative longitudinal data collection curve showed a steady decrease to 88% (representing overall data collection) at day 180. Although cumulative longitudinal data collection with smart bands was 46% at day 90, longitudinal compliance rate for smart bands more rapidly decreased, reaching 17.5% at day 180.

Daily data collection of mobile questionnaires is relatively smaller than the daily data collection of the semiautomated walking steps collection systems. The daily questionnaires consisted of self-reporting modules for daily anxiety, sleep, and emotion questionnaire [6,9], and in total, 20,733 data points were collected via the smartphone app. The overall data collection rate for using the smartphone app self-reporting modules was 75.36% (20,733/27,513). Therefore, we can use the self-reported value of daily anxiety, sleep, and emotion as focal outcome variables to develop distress screening system in our future study. The weekly DT and biweekly PHQ-9 were also collected through the same app, and the overall weekly data collection rates for them were 42.42% (3597/8480) and 41.86% (1775/4240), respectively. As we expected, the self-reported value was less diligently collected than the semiautomated walking step counts.

### Factors Related to Data Collection Rates

In univariate analyses, comorbidities, antihormonal therapy, and targeted therapy were significantly associated with greater data collection rates with the walking app (Table 1). Table 2 shows the results of hierarchical regression analysis. In the first step including demographic variables, no factors were associated with data collection rates for smartphone apps and smart bands. In step 2, we added clinical factors to the model. Since clinical factors such as the types of therapy patients received are important to explain the data collection rate, the amount of variance explained by data collection rates with the app increased to 11.7%, with age, comorbidity, antihormonal therapy, and targeted therapy as significant predictors. Age became a significant factor in step 2 as other factors were included in the model. In step 3, the final model explained 12% of the variance in the data collection rates with the smartphone app. All variables with significance in step 2 remained significant in the final model. Young women were more compliant in the collection of PA data via the smartphone app. Subjects with any kind of disease in addition to breast cancer had a higher rate of data collection with the app (*P*=.001; Table 2). Patients on antihormonal (*P*=.009) or targeted (*P*=.011) therapies had significantly higher rates of data collection with the app (Table 2). In contrast, no demographics or clinical factors were associated with compliance with wearing the smart band (Table 3).

We also tested the correlation between the data collection rates for the use of the app and smart band. The 95% confidence interval ranged from –0.22 to 0.09, and the *P* value was 0.41, indicating there was no correlation between these data collection rates.

**Table 2:** Factors associated with data collection with the smartphone app in multivariate analysis.

Variable	Step 1: demographics (N=160)		Step 2: clinical factors (n=158)			Step 3: depression-related factors (n=158)	
	Beta	*P* value		Beta	*P* value	Beta	*P* value
Constant	1.09	<.001		0.71	.006	0.90	.003
Age	–0.01	.22		–0.01	.04	–0.01	.02
Marital status: single	–0.09	.15		–0.01	.10	–0.09	.14
Marital status: other	0.03	.81		0.11	.41	0.06	.70
≤High school	–0.01	.97		–0.02	.68	–0.02	.62
No occupation	–0.01	.95		0.01	.93	–0.01	.94
Comorbidity: yes	—^a^	—		0.13	.002	0.13	.001
Mastectomy (baseline: breast-conserving surgery)	—	—		–0.11	.25	–0.12	.20
Mastectomy with reconstruction (baseline: breast-conserving surgery)	—	—		–0.11	.10	–0.08	.26
Previous chemotherapy	—	—		0.04	.45	0.04	.49
Antihormonal therapy	—	—		0.14	.01	0.14	.009
Radiation therapy	—	—		–0.08	.25	–0.07	.33
Targeted therapy	—	—		0.17	.02	0.20	.01
Stage I (baseline: stage 0)	—	—		0.03	.62	0.01	.90
Stage II	—	—		–0.04	.58	–0.06	.39
Stage III	—	—		0.10	.25	0.09	.30
EQ5D-5L^b^ index	—	—		0.22	.27	0.31	.14
Distress Thermometer ≥5	—	—		—	—	–0.07	.13
PHQ-9^c^ ≥11	—	—		—	—	0.04	.49
Previous depression: no	—	—		—	—	–0.24	.17
Previous depression: no response	—	—		—	—	–0.18	.39
Adjusted *R*^2^	–0.01	.68		0.12	.005	0.12	.008

^a^Not applicable.

^b^EQ5D-5L: 5-dimension 5-level health questionnaire.

^c^PHQ-9: Patient Health Questionnaire–9.

**Table 3:** Factors associated with data collection with smart bands in multivariate analysis.

Variable	Step 1: demographics (N=160)		Step 2: clinical factors (n=158)		Step 3: depression-related factors (n=158)	
	Beta	*P* value	Beta	*P* value	Beta	*P* value
Constant	0.65	.002	0.94	.02	0.81	.09
Age	–0.01	.81	–0.00	.62	–0.01	.58
Marital status: single	–0.06	.53	–0.07	.47	–0.05	.63
Marital status: other	–0.08	.71	–0.15	.498	–0.18	.49
≤High school	–0.01	.88	–0.01	.98	–0.01	.91
No occupation	–0.07	.26	–0.07	.26	–0.07	.28
Comorbidity: yes	—^a^	—	–0.03	.66	–0.03	.67
Mastectomy (baseline: breast-conserving surgery)	—	—	0.20	.19	0.21	.17
Mastectomy with reconstruction (baseline: breast-conserving surgery)	—	—	–0.15	.17	–0.12	.27
Previous chemotherapy	—	—	0.06	.46	0.07	.46
Antihormonal therapy	—	—	–0.03	.70	–0.02	.83
Radiation therapy	—	—	0.05	.67	0.05	.68
Targeted therapy	—	—	–0.07	.56	–0.05	.68
Stage I (baseline: stage 0)	—	—	0.14	.14	0.12	.19
Stage II	—	—	0.15	.19	0.1	.22
Stage III	—	—	0.01	>.99	–0.01	.92
EQ5D-5L^b^ index	—	—	–0.29	.36	–0.21	.53
Distress Thermometer ≥5	—	—	—	—	–0.07	.29
PHQ-9^c^ ≥11	—	—	—	—	0.11	.17
Previous depression: no	—	—	—	—	0.06	.84
Previous depression: no response	—	—	—	—	0.01	.97
Adjusted *R*^2^	–0.02	.87	–0.01	.51	–0.01	.60

^a^Not applicable.

^b^EQ5D-5L: 5-dimension 5-level health questionnaire.

^c^PHQ-9: Patient Health Questionnaire–9.

### Correlation Between Physical Activity and Patient Distress

In Pearson correlation tests, for smart bands, anxiety, emotion, DT, and the sum of PHQ-9 were negatively correlated with the daily average steps (Table 4). Since these psychological factors were asked to be scored high when the patients felt more depressive, the negative correlation means more daily steps relates with lower distress. The sleep disturbance answer was positively correlated with the daily average steps. Since the sleep disturbance score was asked to be scored low when patients felt unable to sleep, the positive correlation means higher daily steps relates with higher quality of sleep. For the smartphone app, all psychological domains show the same direction of correlation with the daily average steps as for the smart band, although the coefficients were not statistically significant for all domains.

**Table 4:** Correlation between daily steps and examined psychological domains.

Psychological domains	Smartphone app		Smart band	
	Coefficient	*P* value	Coefficient	*P* value
Anxiety (0-10)	–0.07	.38	–0.23	.008
Emotion (0-10)	–0.03	.71	–0.24	.003
Sleep (0-10)	0.08	.36	0.20	.02
Distress Thermometer (0-10)	–0.13	.11	–0.31	<.001
Patient Health Questionnaire–9 sum (0-27)	–0.12	.15	–0.30	<.001

## Discussion

### Principal Findings

Our results indicate that both smartphone app– and smart band–based technologies are feasible tools to collect PA data from breast cancer survivors. The overall data collection rates using both smartphone walking apps and smart bands were higher than the self-reporting rates with smartphone apps in our previous study, despite the longer follow-up period in this study [9]. Although data collection rates with smart bands rapidly decreased over time, PA data from smart bands were significantly correlated with participants’ distress data. Patients who were young, with comorbidities, or receiving antihormonal or targeted therapies were more likely to be adherent to smartphone apps.

To our knowledge, this is the one of the largest prospective studies to assess PA data collection using mobile devices with a longer term of follow-up. In addition, the unique feature of this study is that we enrolled breast cancer patients who are usually recommended to be screened for depressive symptoms, and their depressive symptoms were significantly correlated with PA data from their mobile devices in our study. These results suggest that mobile devices have a significant potential as tools for distress screening in these patients with unmet needs.

Two types of PA data can be collected via smartphone apps. First, self-reported data manually capture health information. For the self-reporting of PA, PA app users self-monitored and recorded exercise more frequently over a 6-month study (2.6 [SD 0.5] days per week) than did non-app users (1.2 [SD 0.5] days per week PA self-monitoring, *P*=.001) [13]. This finding suggested that app-based PA self-reporting is better than non-app–based methods such as paper or Web questionnaires; however, the overall compliance was only 37%. Second, PA data may be obtained from mobile phone sensing via built-in or external sensors. There are a few feasibility studies on the daily collection of PA data through mobile apps or smart bands. One study reported that the number of days with nontypical wear patterns of wearable devices (Fitbit One) ranged from 5% to 9% of all observation points for 5 weeks [14]. Days were marked as nontypical to indicate that the tracker may not have been consistently worn throughout the day and/or data were not recorded, possibly due to a depleted battery. Although the number of missing days was small, the follow-up period was too short. Longer follow-up periods can result in lower compliance rates. Therefore, feasibility studies of daily collection rate of PA through smart bands should be conducted with longer follow-up periods. Our study investigated this issue with more subjects during a longer follow-up period. The results showed that the data collection rates with the smartphone app and smart bands remained higher for 6 months compared with those in other studies.

In terms of cancer survivors, previous studies focused on ePRO data, questionnaires collected via a downloadable app or Web-based portal [15,16]. Authors from a German cancer center reported an adherence rate to a smartphone app–based questionnaire of about 70% in 40 cancer patients over about 100 days [17]. Our previous studies demonstrated an overall compliance rate to an app-based questionnaire of 45% in 30 participants over a 90-day study period [9]. The overall data collection rates with mobile devices in our study were higher than the self-reporting rates in smartphone apps in previous studies.

We found that female breast cancer patients who were young, with comorbidities, or receiving antihormonal or targeted therapies tended to be more adherent to the use of smartphone apps. After enrollment, some patients were administered antihormonal or targeted therapies according to clinical practice guidelines. In terms of antihormonal therapy, patients generally have to take medications daily for 5 to 10 years [18]. Patients administered targeted therapy are usually required to visit the hospital every 3 weeks for approximately 1 year [19]. Patients with other comorbidities may also have to take other medicines or visit the clinic regularly. Thus, one possible explanation is that patients with a regular lifestyle such as taking medicines every day are more likely to check their time schedule with their smartphone, which may lead to increased adherence to smartphone apps. Breast cancer patients are more actively involved in PA collection through smartphone apps because wearing smart bands has caused discomfort to the elderly women. In interviews with patients, we were told that wearing smart bands was inconvenient, and thus, the patients were more active in PA collection through apps than bands.

With early diagnosis and improved treatment, the number of cancer survivors has increased worldwide [20]. Along with the increased cancer survival rates, more attention should be paid to the lives of cancer survivors. Recently, digital footprints, data generated passively through mobile technologies, have been introduced as tools for psychiatric research [10]. Several studies have suggested the potential for the use of data from mobile devices for new measures of mental health [21]. We believe this study can provide future direction to develop distress screening tool using mobile device–based PA data in breast cancer survivors.

### Limitations

Several limitations of this study should be noted. First, long-term adherence to wearable devices has not yet been demonstrated. The follow-up period of this study was 6 months. Second, all subjects in this study were breast cancer patients. Thus, our results can only be generalized to this specific population. Third, all participants were enrolled after the completion of chemotherapy. Therefore, we do not know the feasibility of wearable devices to collect PA data during chemotherapy. Fourth, this study was conducted in a single institution, although the clinical characteristics of the patients in this study were similar to those in a nationwide study [22]. Finally, since we cannot figure out how many hours a day patients were wearing the band and carrying the app, we could not evaluate data quality in terms of time span. Future studies should evaluate the time span of each data collection rather than examining the daily collection of PA.

### Conclusion

Smartphone apps or smart bands are feasible tools to collect daily PA data in breast cancer survivors. PA data from mobile devices are correlated with participants’ distress data, which suggests the potential role of mobile devices in the management of distress in breast cancer survivors. Further research should focus on the interpretation and integration of PA data into clinical practice for the care of breast cancer survivors.

## Abbreviations

DT: Distress Thermometer
ePRO: electronic patient-reported outcome
EQ5D-5L: 5-dimension 5-level health questionnaire
PA: physical activity
PHQ-9: Patient Health Questionnaire–9
PRO: patient-reported outcome
